# Electrophysiology of gliomas: current science, implications, and opportunities

**DOI:** 10.3389/fonc.2025.1611840

**Published:** 2026-01-02

**Authors:** Hanna E. Minns, Nemanja Useinovic, Jordan L. Smith, Sushant Puri, Ahmed M. Raslan, Angelique C. Paulk, Daniel R. Cleary

**Affiliations:** 1 Department of Neurological Surgery, Oregon Health and Science University, Portland, OR, United States; 2 Department of Neurology, Johns Hopkins University School of Medicine, Baltimore, MD, United States; 3 Department of Neurology, Harvard Medical School, Boston, MA, United States; 4 Department of Neurology, Center for Neurotechnology and Neurorecovery, Massachusetts General Hospital, Harvard Medical School, Boston, MA, United States; 5 Department of Surgery, Portland Veterans Affairs Medical Center, Portland, OR, United States

**Keywords:** glioma, glioblastoma, electrophysiology, neuron-glioma interactions, brain mapping, high density electrodes, neuromodulation

## Abstract

Gliomas engage in bidirectional communication with neurons, promoting hyperexcitable conditions that enable neural circuit infiltration and drive tumor growth. These neuron-glioma interactions create patterns of aberrant neural activity that can be detected using intracranial electrodes. While conventional clinical electrodes are limited by low spatiotemporal resolution and lack of single-unit precision, recent advances in neural engineering have introduced multiple types of high-density electrodes that provide orders of magnitude greater spatial resolution. Pairing these tools with emerging characterizations of novel, glioma-associated electrophysiological signatures offers new opportunities to understand disease progression and improve surgical and medical management for gliomas and glioma-related epilepsy. In this review, we begin by outlining foundational research in cancer neuroscience and neuron-glioma interactions through the lens of extracellular dynamics. We then discuss established and emerging methods for intraoperative evaluation of neural activity, what is known about glioma-associated oscillatory and aperiodic trends, and implications for future studies. Finally, we consider the therapeutic potential of neuromodulation for gliomas.

## Introduction

Malignant primary brain tumors – of which more than 80% are gliomas - are responsible for over 15,000 deaths per year in the United States alone (1). Over the past ten years, strides have been made to uncover the electrophysiological underpinnings of gliomas and to understand their contribution to epilepsy. Glioma cells are derived from neural and glial precursors and, as such, are uniquely positioned to manipulate the functions of their healthy counterparts. We now know that glioma cells communicate with innate cell types of the central nervous system, which drives tumor growth and invasion, as well as seizure activity (2, 3). A growing body of work also shows that glioma cells integrate into physiologically active neural circuits (4–7). Despite these advances in understanding, this knowledge has yet to significantly change surgical or medical management (8).

Given that gliomas dysregulate neural activity at the single-cell level, glioma-specific electrophysiological patterns can also be detected clinically. Imaging studies have lent insight into tumor-induced plasticity and alterations in functional connectivity such as contralateral compensation networks (9, 10), but imaging remains intrinsically limited by indirectly measuring neural activity. Direct electrophysiological recordings – such as those used during intraoperative intracranial brain mapping – provide a more accurate and detailed assessment of the physiological impact of glioma infiltration. With advances in the spatiotemporal resolution of recording electrodes, opportunities exist to uncover tumor-associated electrophysiological patterns. These discoveries could improve patient care by refining surgical approaches, identifying epileptic drivers, and guiding the development of treatments that target tumor-neuron interactions.

Understanding this pathological electrical activity could also expose new avenues for therapeutic neuromodulation of gliomas. While pharmacological targeting with neuroactive drugs has garnered momentum, efficacy in the form of randomized clinical trials has yet to be seen. Non-pharmacological neuromodulation using bioelectronic devices may offer an alternative or complementary strategy. However, an increased understanding of their effect on neuron-glioma circuits is needed in order to inspire therapeutic design and clinical frameworks. In this review, we provide an overview of 1) neuron-glioma crosstalk and contributions to extracellular electrical currents, 2) intraoperative detection of glioma electrophysiology and novel approaches to brain mapping, and 3) neuromodulation for targeting dysfunctional glioma circuits.

## Neuron-glioma crosstalk and contributions to extracellular electrical currents

**Figure 1:**
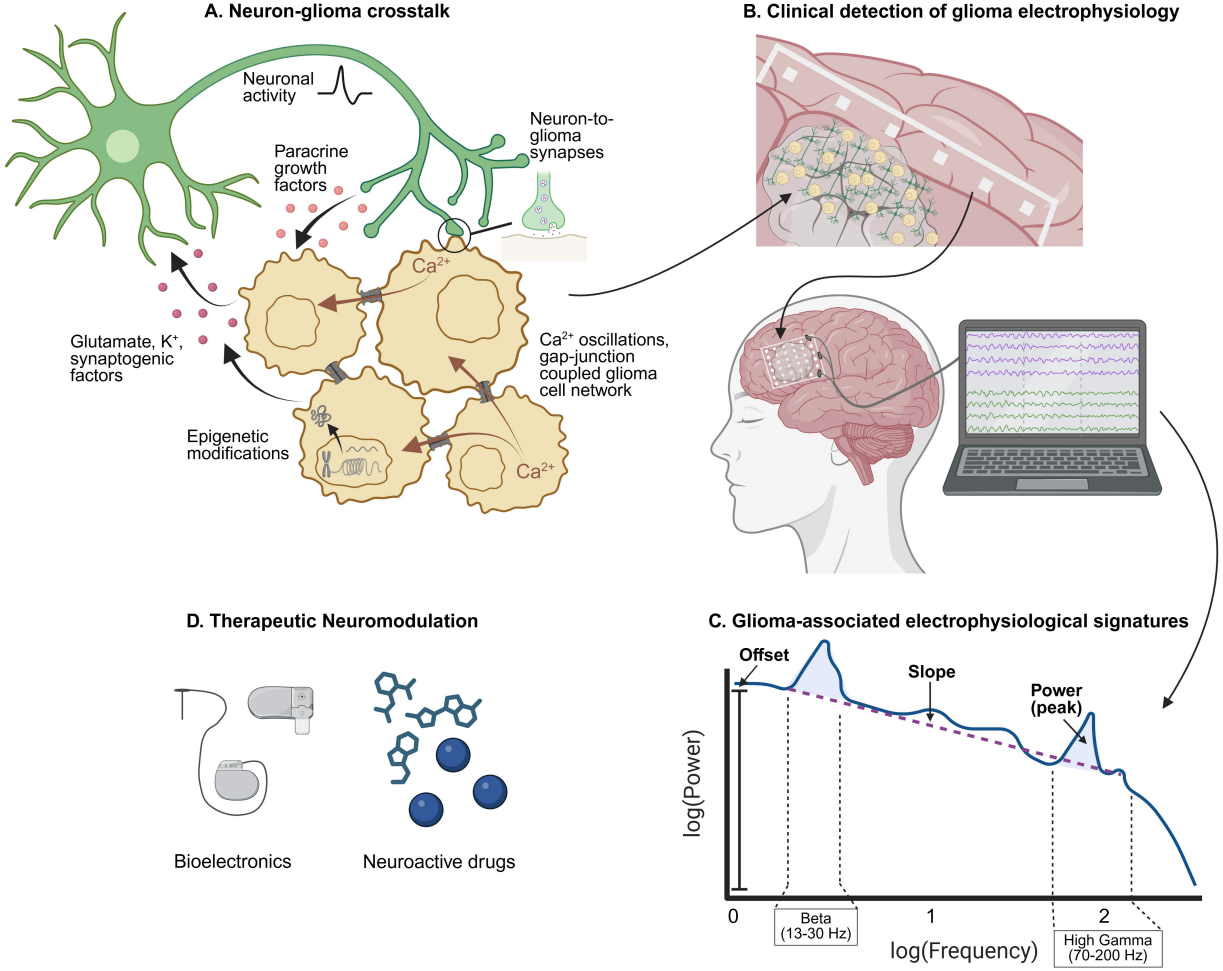
Glioma electrophysiology. **(A)** Neuron-glioma crosstalk and glioma cell networks drive tumor progression through various mechanisms that concurrently affect local field potential. Adapted from *The Neuroscience of Cancer* (2). **(B)** Dynamic neuron-glioma interactions aggregate into large scale electrophysiological signatures that can be recorded using intracranial electrodes. **(C)** Intracranial recordings can be deconvoluted to reveal glioma-associated oscillatory and aperiodic trends that can be visualized using the power spectrum. **(D)** Increased understanding of glioma-associated electrical activity will reveal new avenues for therapeutic neuromodulation.

Pioneering work over the past decade has outlined how bidirectional neuron-glioma communication encourages tumor growth through activity-dependent paracrine factors, direct electrochemical synapses, and glioma cell-intrinsic electrical properties (**Figure 1A**) (2, 3). Although these findings define the pathophysiological mechanisms of gliomas at the single cell level, for the development of global electrical phenomena such as epilepsy – or perhaps widespread tumor infiltration - to occur, changes must be happening more broadly. In addition, existing clinical methods for detecting neural activity most often reflect larger-scale patterns as measurements of local field potential (LFP). LFP are aggregate electrical signals that reflect both local neuronal activity as well as regional or global fluxes (11). As such, LFP is influenced not only by shifts in electrical currents, but also by context-dependent factors such as the geometry of current sources, the spatial and temporal scale of regional network synchrony or lack thereof, cognitive state, tumor type, and cortical versus subcortical areas (11–16). To understand the full electrophysiological effects of glioma invasion, the cellular mechanisms of neuron-glioma crosstalk should be considered with regards to their local contributions to extracellular ionic flow as well as broader physiological changes.

### Neuron-to-glioma communication and glioma-cell-intrinsic mechanisms

Direct neuron-to-glioma synapses exist and result in excitatory post-synaptic currents (EPSCs), glioma cell depolarization, and tumor proliferation (17, 18). This pathological integration was first shown with glutamatergic synapses (17, 18), but recent work shows that glioma cells can receive GABAergic (19), cholinergic (20), serotonergic, adrenergic, and dopaminergic synaptic inputs (6, 7), allowing them to infiltrate a variety of brain-wide circuits. By communicating with a diversity of neuronal subpopulations, gliomas are highly adaptable, driving tumor progression and invasion throughout the brain while simultaneously complicating LFP interpretations, impeding therapeutic targeting and contributing to a myriad of neurological symptoms that affect patients’ quality of life.

Glioma cells themselves produce non-synaptic, activity-dependent potassium currents with low input resistance, similar to those made by normal glial cells, as well as autonomous rhythmic calcium-based oscillations generated from a small subset of “pacemaker-like” glioma cells (17, 21). These electrochemical signals then spread through a synchronized, gap-junction coupled tumor cell network (18, 22, 23). Many studies have also implicated activity-dependent secretion of synaptic regulator proteins in the ability of glioma cells to form synapses, as well as in proliferation and invasion (5, 24–28). In addition, other groups have highlighted epigenetic mechanisms that facilitate neuron-glioma interactions including hypomethylation with an upregulation of synaptic (29) and neuroactive ligand-receptor interaction genes (30). Extensive chromatin remodeling has also been shown to sustain neuron-glioma synapses through enhancer rewiring and transcription factors that regulate synaptic organization and axon guidance genes (31). Furthermore, glioma cells receive and respond to afferent neuronal inputs from a variety of local and far-ranging sources. They also contain intrinsic mechanisms and regulatory networks that bolster communication with neurons. All of which not only drives tumor progression but also dynamically influence extracellular current measurement at any given moment.

### Glioma-induced neuronal hyperexcitability

In the opposite direction, glioma cells induce neuronal hyperexcitability through mechanisms such as aberrant glutamate transport and impaired inhibitory signaling (32–37). Inhibitory neurons are not lost with gliomas but are instead recruited to participate in excitatory activity (38). Further, GABAergic signaling, which is typically thought of as inhibitory through cell hyperpolarization, can instead become depolarizing (19). One explanation for this is that many gliomas are thought to originate from oligodendrocyte precursor cells (OPCs) (39, 40), and normal GABAergic neuron-to-OPC synapses during development can be excitatory due to high intracellular chloride (41). In addition, OPC-like glioma cells are important drivers of epileptic activity at the leading edge via dysregulation of voltage-gated potassium channels leading to increased extracellular potassium and neuron depolarization (42, 43), as well as upregulation of synaptic gene expression programs and increased colocalization with neurons (30). Lastly, there are subtype-specific propagators of hyperexcitability such as increased levels of D-2-hydroxyglutarate produced by IDH mutated gliomas (44), a mechanism that IDH wildtype tumors may be largely insensitive to (45), and certain PIK3CA driver variants (46). These subtype-specific alterations may allude to why different types of gliomas, such as IDH mutant tumors, have higher rates of glioma-related epilepsy (44, 47, 48).

This imbalance between excitatory and inhibitory synaptic activity (E/I) promotes seizure-like events (SLEs) at the tumor margin and peritumoral regions, whereas the tumor core remains electrically silent (17, 34). SLEs can consequently develop into glioma-related epilepsy (GRE) - a common and often refractory symptom of gliomas (49). Ultimately, this reprogramming of canonical inhibitory signaling may represent an escape mechanism for tumor cells seeking to exploit neuronal activity-regulated factors. Glioma-induced neuronal excitability can further drive activity-dependent mechanisms of tumor growth and invasion, feeding a malignant cycle. Although the distorted E/I dynamics challenge treatment strategies and traditional electrophysiological analyses, tumor-associated E/I imbalances may be informative at a greater scale using intraoperative recordings (50–53), as discussed in the next section.

Neuron-to-glioma communication, synchronous glioma-cell networks and glioma-induced neuronal activity drive tumor progression and likely alter field potentials simultaneously. LFP interpretations are further confounded by the heterogeneous structural, spatial, and temporal relationships between the underlying current sources and sinks, especially as infiltrative gliomas such as GBM must be thought of as whole brain diseases. A less ambiguous approach may be to explore the fundamental changes in excitability at the level of the single neuron using single-unit recordings, which have become more feasible in recent years.

## Clinical detection of glioma-associated electrophysiological signals

### Methods of detecting neural activity

The modalities for measuring LFP in clinical settings include scalp EEG, cortical surface electrocorticography (ECoG, **Figure 1B**), and intracortical stereoencephalography (sEEG) (54). Subdural recording devices can be used in combination with direct electrical stimulation (DES) for brain mapping (use of electrical stimulation to disrupt function) during intraoperative resections of tumors situated in or near eloquent regions. Although LFP can be measured using any number of intracranial electrodes, clinical macroelectrodes have the advantage of low impedance, wider spatial coverage, handling ease, and FDA approval. While standard brain mapping techniques using clinical macroelectrode grids currently help avoid surgical damage to functional areas, they have limits.

**Figure 2:**
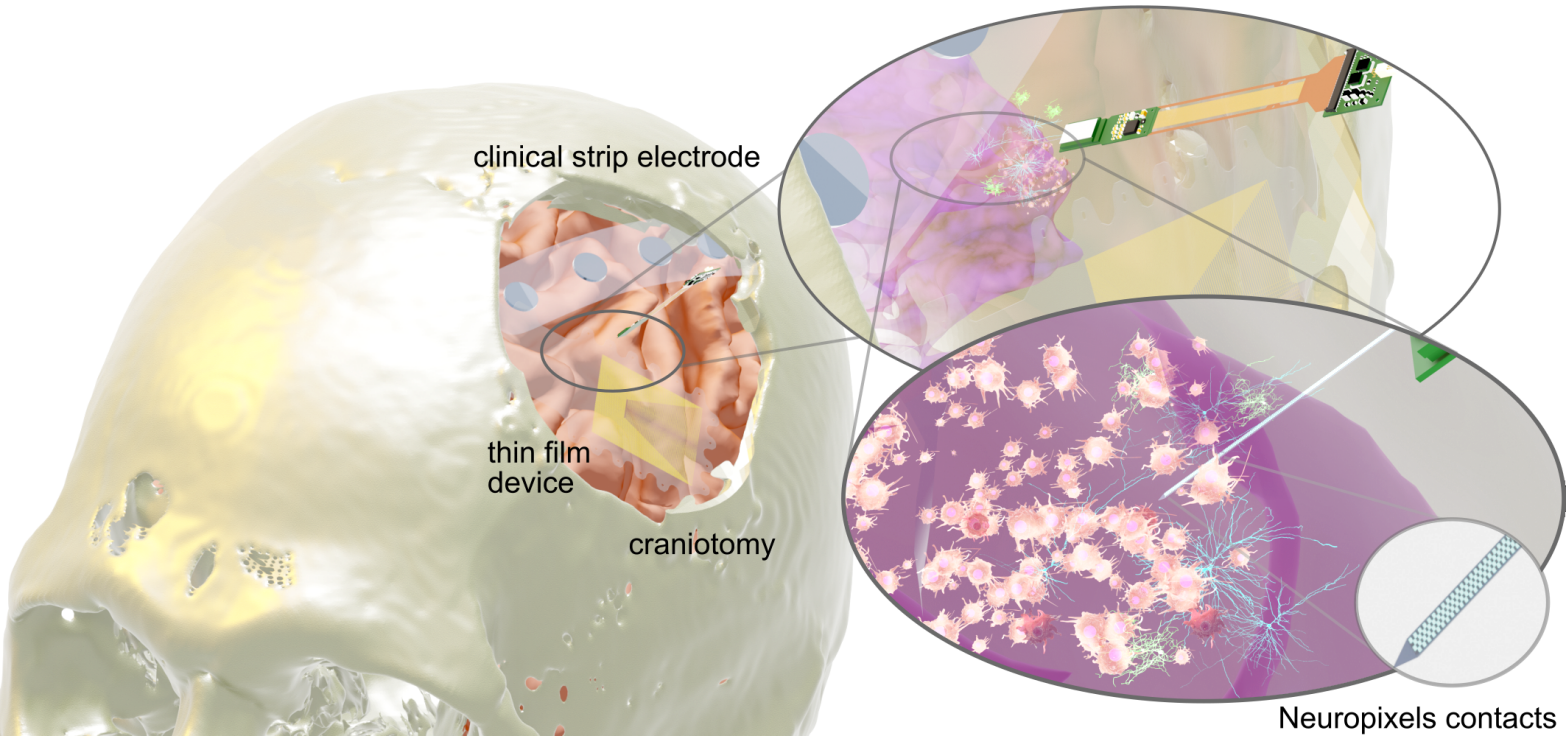
Intracranial recording electrodes. Left, from top to bottom: conventional clinical strip electrode, Neuropixels probe, high-density thin-film grid. Right: representation of how Neuropixels probe captures single-unit activity between neurons and glioma cells.

Most conventional clinical electrodes have a diameter of 4mm and a pitch of 1cm, which averages electrophysiological signal over a relatively large area of cortex while still leaving a significant area uncovered. As a result, they lack the spatial resolution for localizing coordinated hubs of neural activity (55). Also, increasing distance between the recording electrode and current sources directly reduces signal quality, thus limiting the utility for tumors situated anywhere but directly under the grid (12). This effect is attributable to signal attenuation and dispersion, as well as further amplification of context-dependent confounders (e.g. spatial orientation, neuronal morphology) when recording from larger populations of neurons, all of which result in lower signal-to-noise ratio of recordings. Since electrodes are not useful for recording signals from white matter, neural recordings cannot help in many scenarios of deep invasion, which is common given that gliomas often invade along axon tracts (56). These limitations highlight that existing methods for detecting neural activity come with a tradeoff between precision and spatial coverage. Although FDA-approved ECoG grids are the standard-of-care, novel research electrodes are currently being developed to provide finer resolution while maintaining the ability to record from clinically significant spatial areas. For example, both Neuropixels probes and high-density thin-film electrodes have already demonstrated superior resolution for functional localization during brain mapping (**Figure 2**) (57–60). With the ability to characterize specific waveforms and finely track regional spike propagations, these higher resolution electrodes could contribute significantly to our understanding of glioma electrophysiology.

### Power spectrum outputs

Even with traditional ECoG, patterns of electrical activity produced by physiological and pathological states can be detected through oscillatory and aperiodic components of the power spectrum (**Figure 1C**) (61). Oscillatory components are typically thought of in relation to canonical frequency bands. In the normal brain, high frequency signals like in the high gamma range (typically >70 Hz and <200 Hz) exhibit discrete spatial localizations and have been attributed to fast spiking sub-populations, such as those activated in task-dependent circuits (62–64). In contrast, lower frequencies tend to be more spatially diffuse and have been interpreted as slower fluctuations of synchronous inputs such as those modulating attention, memory, or perception (55, 65–67). These frequency-specific oscillations are represented as peaks of power above the aperiodic component (68). The aperiodic component, in contrast, represents non-oscillatory activity and can be assessed with parameters such as the offset and slope (68). The offset represents a uniform shift in power across frequencies and is correlated with increased neuronal spiking activity, whereas the slope of the aperiodic component has been shown to reflect the E/I balance, with a steeper slope indicating a lower E/I ratio (increased inhibitory tone) and a flatter slope indicating a higher E:I ratio (increased excitatory tone) (69).

In some cases, glioma-related electrophysiological trends are diffuse and non-specific to tumorigenesis, mirroring those seen in other brain pathologies such as increased slow-wave activity observed in stroke patients and increased high frequency oscillations in glioma-related epilepsy (33, 70, 71). However, recent work demonstrates the possibility of more specific tumor-associated signals such as altered E/I balance reflected in the aperiodic slope (15, 72, 73), frequency-specific changes that delineate peritumoral versus intratumoral regions (73), and task-dependent high gamma alterations within well-defined functional circuits (4, 5, 74–76). Further, given that brain mapping is commonly employed intraoperatively for tumor resections, these signatures represent promising biomarkers to more precisely identify and characterize pathological tissue.

### Glioma-associated aperiodic alterations

Recent studies have begun to explore the relationship between the aperiodic slope and different factors of glioma pathology (15, 72, 73, 77). Using intraoperative ECoG recordings, one group found that in higher frequency bands (gamma: 30–50 Hz and high gamma: 70–150 Hz), glioma-infiltrated cortex had a flatter aperiodic slope compared to normal-appearing cortex, indicating an excitation-dominant state (77). They also found a positive correlation between these flatter aperiodic slopes and increased tumor cell infiltration, glutamatergic gene expression, and tumor grade, with IDH-wildtype GBMs, the most aggressive glioma subtype, displaying the flattest aperiodic slope (77). Other studies have explored the aperiodic slope over frequencies below 50 Hz, with one showing that IDH-wildtype GBMs preferentially occur in brain regions with intrinsically steeper aperiodic slopes (lower E/I ratio, inhibitory-dominant state) as measured from healthy participants (15). Peritumoral regions have also shown steeper slopes than intratumoral regions in the 20–40 Hz range (73).

These findings highlight that the interpretation of aperiodic slope is highly dependent on the frequency range considered (78), emphasizing the need for systematic characterization across bands. However, across studies, a consistent observation remains an increase in offset, and frequency-specific power as discussed below, in glioma-infiltrated tissue outside of the tumor core, despite molecular subtype, suggesting an overall elevation in neuronal activity (15, 72, 73, 79). Taken together, these data indicate that while general increases in neuronal activity are a common feature of gliomas, variation in E/I balance reflected by the slope may help stratify molecular subtypes, distinguish tumor boundaries, and provide further insight into evolving tumor dynamics.

### Task-dependent, glioma-associated alterations of well-defined functional circuits

Most studies so far focused on task-dependent alterations of canonical language circuits in patients with gliomas infiltrating dominant, left frontal cortical language areas (4, 5, 74, 75). Using intraoperative ECoG, differences in high gamma power (HGp) between normal-appearing and glioma-infiltrated cortex have been investigated during audiovisual naming tasks (4, 5). HGp is closely associated with increased spiking activity and neuronal synchrony (64). At sites with normal-appearing brain, ECoG recorded a task-dependent increase in HGp that was spatially confined to known areas of speech planning and initiation. At glioma-infiltrated sites, activation was also task-dependent, but maximal HGp increased more diffusely, including recruitment of non-canonical language areas. These differences are echoed by task-based fMRI (tb-fMRI) studies of syntax networks where healthy participants had task-dependent activation of three separate syntax networks with little cross-talk between networks, while glioma patients showed a lack of network boundaries with increased inter-network activity (74, 75). For motor tasks, ECoG has been used to record task-dependent differences of finger or wrist exercises in patients with motor cortex glioma (MCG) (76). In non-MCG patients, individual fingers had distinct locations within the motor cortex and individualized responses to direct electrical stimulation, but in MCG patients, these distinct spatial representations were lost and replaced instead by diffuse arrangements and remodeled activation patterns upon stimulation. Moreover, neural activity in glioma-infiltrated regions is clearly task-relevant and physiologically organized. However, gliomas also induce pathologic circuit remodeling with more diffuse high gamma activity and synchrony with other nearby areas.

Language studies have also been used to examine the effects of glioma infiltration on cortical activity during increasingly difficult tasks, such as monosyllable versus polysyllable conditions (4, 5). In normal-appearing cortex, the more difficult tasks elicited an upregulation of HGp which was not detectable when recording from glioma-infiltrated regions. Recordings from glioma regions produced below-chance decoding of the easier versus harder word trials compared to normal regions. Comparably, harder syntactic load elicited significantly decreased activation of syntax networks on tb-fMRI in glioma patients compared to healthy controls (74, 75). Taken together, this suggests that hyperconnected glioma-to-glioma and neuron-to-glioma networks may limit the ability to perform selective and specific processes at the local level, thus reducing the ability to coordinate more dynamic behavioral responses. Similar pathophysiology is seen in Alzheimer’s Disease (AD) patients, for example, where AD-associated cognitive impairment correlates with neuronal hyperexcitability and hyper-synchronization of brain regions (80–82). Indeed, gliomas are known to cause cognitive impairment (83, 84), and some studies have linked this with higher functional connectivity on imaging and scalp EEG (85, 86). Furthermore, conditions of increased task complexity may be crucial to revealing abnormal patterns intraoperatively (87).

In summary, recent work demonstrates that gliomas produce altered patterns of neural activity that can be detected intraoperatively using ECoG. However, current limitations include the difficulty of rigorous intraoperative studies, incomplete comparisons between glioma subtypes, anatomical regions, and between primary versus recurrent tumors, and importantly, the lack of spatial recording methods that match cortical physiology. Leveraging novel detection methods will allow more precise characterization of electrophysiological biomarkers to improve understanding and management of this disease.

## Future directions: targeting glioma electrophysiology with neuromodulation

Aberrant electrical phenomena of glioma invasion could be targeted using pharmacological and non-pharmacological neuromodulation (**Figure 1D**). Pharmacological neuromodulation has been shown to reduce glioma growth in preclinical models with anti-epileptic drugs such as gabapentin (5) and perampanel (17, 18), which block synaptogenic factor TSP-1 and excitatory glutamate receptors (AMPA receptors), respectively, as well as using ADAM10 inhibitors to prevent the cleavage of synaptic adhesion molecule and oncogenic protein neuroligin-3 (25). These findings provided a foundation for numerous clinical trials exploring the efficacy of neuroactive drugs for glioma treatment, most of which are ongoing. These include perampanel (88), gabapentin, sulfasalazine, and memantine (89), levetiracetam (90, 91), valproic acid (92), and ADAM10 inhibition with INCB7839 (93). While some retrospective studies have correlated anti-epileptic drug use with survival benefit in glioma patients (94–99), others concluded conflicting results (90, 100–103). Ultimately, the efficacy of pharmacological neuromodulation for glioma treatment is inconclusive as more randomized clinical trials are needed (104).

Given the range of mechanisms sustaining neuron-glioma crosstalk and the diversity of neuronal subpopulations and neurotransmitters involved, progress will likely require combination or multimodal approaches. Non-pharmacological neuromodulation using bioelectronic devices may offer a complementary therapeutic avenue. While current devices used to treat other diagnoses of dysfunctional neural circuits may provide a useful framework (105, 106), it will be important to understand the specificity of potential technologies for glioma-associated neural interactions, without adversely affecting normal physiology (107). Furthermore, an increased understanding of glioma electrophysiology facilitated by higher resolution detection methods and intraoperative studies will help establish a framework for designing personalized neuromodulatory devices for the treatment of gliomas.

## Conclusion

Glioma cells communicate with normal neurons and glial cells, driving hyperexcitability, infiltration into local and brain-wide networks, and tumor progression. Neurosurgical and technological advances have facilitated detection and targeting of pathological neural activity at a clinical level. Characterizing glioma electrophysiology has the potential to better inform mechanisms of glioma growth and glioma-related epilepsy, enhance intraoperative brain mapping and refine neuromodulation strategies. This review provides an overview of current knowledge as well as limitations pertaining to our understanding and interpretation of glioma electrophysiology. By providing a roadmap from single neuron-glioma communication to detection and targeting of summative electrical activity, we highlight promising directions for improving patient care and glioma outcomes.
